# Supplementary material for: Evaluation of weight retention four weeks after delivery as a risk factor for gestational diabetes mellitus in a subsequent pregnancy
Source: PLoS One. 2020 Apr 2;15(4):e0231018. doi: 10.1371/journal.pone.0231018 (PMC7117671; doi:10.1371/journal.pone.0231018)
Supplement: Attachment — Submitted filename: Response to Reviewers.docx [file pone.0231018.s002.docx]

March 5, 2020

Zhong-Cheng Luo

Academic Editor

*PLOS ONE*

Dear Editor:

I, along with my co-authors, would like to re-submit the attached manuscript, titled “Evaluation of weight retention four weeks after delivery as a risk factor for gestational diabetes mellitus in a subsequent pregnancy” as an original research article (manuscript ID: PONE-D-19-31440).

We appreciate your valuable comments, which have been very useful to us in improving the quality of our manuscript. All changes are shown in red in the revised manuscript. The entire manuscript has been also rechecked, and the necessary changes have been made in accordance with your suggestions. Our point-by-point responses to all of your comments have been prepared and provided below.

We believe that the changes made based on your comments have significantly improved our manuscript, and we hope that you will find it suitable for publication in *PLOS ONE*.

Sincerely,

Dr. Satoshi Shinohara

Department of Obstetrics and Gynecology

National Hospital Organization Kofu National Hospital

11-35 Tenjin, Kofu, Yamanashi 400-8533, Japan

Tel: +81-55-253-6131

Fax: +81-55-251-5597

Email: shinohara617@gmail.com

**RESPONSES TO THE EDITOR’S COMMENTS**

**Comment 1**

Please add an ad hoc power calculaition for detecting a 2-times risk elevatation in GDM comparing postpartum BMI change >=1 (unstable) vs <1 (stable) in the Discussion. You observed almost 2 times risk elevation (adjusted OR=1.93), although it was not statistically significant. This may be due to the lack of or insufficient study power to detect a risk difference. If this is true, data interpretation should be adjusted for accordingly.

**Response 1**

Thank you for your comment. We reviewed the sample size again according to your comments. When performing logistic analysis like this study, it seems that the sample size is often determined based on events per variable (EPV) rather than per ad hoc power calculation (A-C). Calculating the required sample size based on the previous report (10 EPV is a widely adopted minimal guideline criterion for performing logistic regression analysis), we needed at least 60 GDM women during the second pregnancy in this study. Therefore, since this study only included 42 GDM women during the second pregnancy, there is a possibility of the lack of or insufficient study power to detect a risk difference as pointed out. Therefore, we added in the limitation section; “According to the sample size calculation method widely used when performing logistic analysis, “Ten Events Per Variable” is a widely adopted minimal guideline criterion for performing logistic regression analysis. Based on this method, we needed at least 60 GDM women during the second pregnancy in this study. Since this study only included 42 GDM women during the second pregnancy, there is a possibility of the lack of or insufficient study power to detect a risk difference.”

A) Peduzzi P, Concato J, Kemper E, Holford TR, Feinstein AR. A simulation study of the number of events per variable in logistic regression analysis. J Clin Epidemiol. 1996;49: 1373-1379.

B) Moons KG, de Groot JA, Bouwmeester W, Vergouwe Y, Mallett S, Altman DG, et al. Critical appraisal and data extraction for systematic reviews of prediction modelling studies: the CHARMS checklist. PLoS Med. 2014;11: e1001744.

C) Moons KG, Altman DG, Reitsma JB, Ioannidis JP, Macaskill P, Steyerberg EW, et al. Transparent reporting of a multivariable prediction model for individual prognosis or diagnosis (TRIPOD): explanation and elaboration. Ann Intern Med. 2015;162: W1-W73.

**Comment 2**

Please add a cloumn for the accurate P value to the precision of 3 decimal points for

the adjusted OR in Table 2.

**Response 2**

Thank you for your comment. Following your comment, we revised Table 2.

**Comment 3**

Line 184, why stated "(data not shown)" when you acturally presented the data?

**Response 3**

Thank you for your comment. According to your comment, we added Table 3, and “Finally, a

multiple logistic regression model, into which we entered PPWR as a continuous variable,

was performed. After controlling for each variable, a history of previous GDM, and obesity

were significantly associated with GDM in the second pregnancy (history of previous GDM:

adjusted OR, 7.70; 95% CI, 3.08–19.2; obesity: adjusted OR, 4.13; 95% CI, 1.91–8.95) in

the multivariable logistic regression analysis. PPWR was not significantly associated with

GDM in the second pregnancy (adjusted OR, 1.19; 95% CI, 0.94–1.50) (Table 3).” in the

result.

**RESPONSES TO THE REVIEWER 1’S COMMENTS**

**Comment 1**

Was the pre-pregnancy weight for calculation of the BMI at the first visit self-reported?

**Response 1**

Thank you for your comment. The pre-pregnancy weight for calculation of the BMI at the first

visit was self-reported. We added, “The pre-pregnancy weight for calculation of the BMI at

the first visit was self-reported.” in the data collection.

**Comment 2**

How was the screening for GDM performed? Was it a 1-step or was there pre-screening

with random BSL or GCT as both of these have been shown to miss up to 20% of cases

of GDM? Understanding this is important for the non-Japanese reader.

**Response 2**

Thank you for your comment. We added “In Japan, an initial screening test followed by the

diagnostic oral glucose tolerance test (OGTT) in screen-positive women, known as the two-

step approach, is used for GDM diagnosis [16]. Moreover, the Japan Society of Obstetrics

and Gynaecology has recommended the 50 g glucose challenge test (GCT) as a screening

method for GDM [16]. Women with abnormal GCT results (serum glucose levels ≥140 mg/dL)

subsequently undergo a 75-g OGTT for a definitive diagnosis.” in the data collection.

**Comment 3**

You have made comment in your discussion that you were measuring weight retention at 4

weeks postpartum that may not accurately reflect the final post pregnancy return to the non-

pregnant state. It was not clear if you have made adjustment for the gestational length of the

pregnancy as this may have a significant impact on the total weight gain and therefore the

peak level from which the woman has to lose weight in returning to the non-pregnancy state.

**Response 3**

Thank you for your comment. Following your comment, we added an analysis of the gestational length of the first pregnancy in Table1.

**RESPONSES TO THE REVIEWER 2’S COMMENTS**

**Comment 1**

A Stable PPWR (4 wk BMI – 1st pregnancy prepregnancy BMI) < 1 BMI unit means that the

individual lost almost all of their pregnancy weight gain by 4 weeks, correct? The definition

of non-stable PPWR (gain of > 1 BMI unit) is only defined in the Abstract and should again

be defined in the Methods and repeated in the results.

**Response 1**

Thank you for your comment. According to your comment, we changed from “We classified

women according to their postpartum check-up PPWR and defined a stable PPWR as ≤1

BMI unit, per previous relevant publications” to “We classified women according to their

postpartum check-up PPWR and defined a stable PPWR as ≤1 BMI unit and non-stable

PPWR >1 BMI unit, per previous relevant publications” in the Methods. Moreover, we

changed from “The characteristics of women with and without were similar except for greater

first and second pregnancy, pre-pregnancy BMIs and lower first pregnancy, postpartum

checkup BMIs in the stable PPWR group (Table 1).” to “The characteristics of women with a

stable (≤1 BMI unit) and a non-stable PPWR (>1 BMI unit) were similar except for greater

first and second pregnancy, pre-pregnancy BMIs and lower first pregnancy, postpartum

checkup BMIs in the stable PPWR group (Table 1).”

**Comment 2**

They found no difference in the risk for GDM according to their separation of groups

according to BMI change at this 4 week follow-up visit, at which only about 1/3 of women had

come back to < 1BMI difference from their prepregnancy BMI. They did find that obesity and

interpregnancy weight gain, as expected, were risk factors for GDM. Could the authors

analyze the PPWR among those who were obese compared to those not obese? Probably

not, as the proportion who were obese was very low (71/566). The authors could mention in

their Discussion, that given the current results, attention should be paid to women with the

traditional risk factors of obesity, weight gain during pregnancy, prior history of GDM and

family history of GDM rather than PPWR – especially at 4 weeks

**Response 2**

Thank you for your comment. According to your comment, we added “According to the

current study results, attention should be paid to women with the traditional risk factors of

obesity, the interpregnancy BMI change, prior history of GDM and family history of GDM

rather than PPWR – especially at four weeks after delivery.” and “In this study, there was no

significant difference in PPWR between obese and non-obese women (1.20±2.29 vs.

1.53±1.28 BMI unit, p=0.51)” in the discussion.
